# Fabrication of silver nanoparticles-deposited fabrics as a potential candidate for the development of reusable facemasks and evaluation of their performance

**DOI:** 10.1038/s41598-023-28858-9

**Published:** 2023-01-28

**Authors:** Morteza Abazari, Safa Momeni Badeleh, Fatemeh Khaleghi, Majid Saeedi, Fakhri Haghi

**Affiliations:** 1 Department of Pharmaceutical Nanotechnology, School of Pharmacy, Zanjan University of Medical Sciences, Zanjan, Iran; 2 Department of Food and Drug Control, School of Pharmacy, Zanjan University of Medical Sciences, Zanjan, Iran; 3 The Health of Plant and Livestock Products Research Center, Mazandaran University of Medical Sciences, Sari, Iran; 4 Pharmaceutical Sciences Research Center, Haemoglobinopathy Institute, Mazandaran University of Medical Sciences, Sari, Iran; 5 Department of Pharmaceutics, Faculty of Pharmacy, Mazandaran University of Medical Sciences, Sari, Iran; 6 Department of Microbiology, School of Medicine, Zanjan University of Medical Sciences, Zanjan, Iran

**Keywords:** Health care, Medical research, Chemistry, Materials science, Nanoscience and technology

## Abstract

Recently, wearing facemasks in public has been raised due to the coronavirus disease 2019 epidemic worldwide. However, the performance and effectiveness of many existing products have raised significant concerns among people and professionals. Therefore, greater attempts have been focused recently to increase the efficacy of these products scientifically and industrially. In this respect, doping or impregnating facemask fabrics with metallic substances or nanoparticles like silver nanoparticles has been proposed. So, in the present study, we aimed to sonochemically coat silver nanoparticles on the non-woven Spunbond substrates at different sonication times and concentrations to develop antibacterial and antiviral facemask. The coated substrates were characterized using Field Emission Scanning Electron Microscope, Energy Dispersive X-Ray, X-ray diffraction, and Thermogravimetry analysis. The amount of silver released from the coated substrates was measured by atomic absorption spectroscopy. The filtration efficiency, pressure drop, and electrical conductivity of the coated samples were also investigated. The antibacterial activity of fabrics was evaluated against *Escherichia coli* and *Staphylococcus aureus*. Cellular viability of samples assessed by MTT and brine shrimp lethality tests. The results revealed that the higher sonication times and precursor concentrations result in a higher and more stable coating, larger particle size, wider particle size distribution, and lower content of released silver. Coated fabrics also revealed enhanced filtration efficiency (against nanosize particles), desired pressure drop, and antibacterial activity without significant cytotoxicity toward HEK 293 cells and Artemia nauplii. As a result, the coated fabrics could find potential applications in the development of facemasks for protection against different pathogenic entities.

## Introduction

Recently, considering the coronavirus disease 2019 (COVID-19) pandemic caused by the SARS-CoV-2 coronavirus around the globe, the use of personal protective equipment (PPE) such as facemasks, protective clothing, hospital clothing, etc. has attracted a lot of attention to protect the wearer and others against the disease and stop it’s spreading^1–3^. However, the performance and effectiveness of many of these products have raised controversial concerns among people and professionals. For example, the maximum fitted filtration efficiency (FFE) of N95 respirator, surgical masks with ties, two-layer woven nylon masks, cotton bandana, single-layer woven polyester gaiter, single-layer woven polyester/nylon masks with ties, non-woven polypropylene (PP) mask with fixed ear loops, and three-layer knitted cotton mask with ear loops are reported about 98.4%, 71.5%, 79.0%, 49.9%, 37.8%, 39.3%, 28.6%, and 26.5%, respectively. Therefore, only N95 respirators provide appropriate FFE against airborne particles^4^. However, it is reported that the N95 filtering facepiece respirators may not protect against small photogenic microorganisms such as bacteria, viruses, etc^5^. Therefore, major efforts have been made recently to increase the efficacy of respirator devices scientifically and industrially for adequate protection of the wearer against pathogenic entities like bacteria and coronavirus. In this regard, endowing antimicrobial and antiviral properties to these products has attracted particular attention.

Numerous compounds have been used to prevent and manage infectious diseases caused by pathogenic microorganisms^6,7^. However, the effectiveness of many of these compounds and products has been restricted due to the outbreak of unknown diseases such as COVID-19 and emerging of antibiotic and multidrug-resistant bacteria and other pathogenic microorganisms. Therefore, dealing with these diseases has become a global dilemma. So, searching for new and effective compounds with a broad or specific antimicrobial and antiviral spectrum and lower toxicity has recently accelerated^8,9^. In the present scenario, nanotechnology-driven products with enhanced surface area-to-volume ratio offer unique physical and chemical properties for different purposes such as antibacterial and antiviral applications^10–12^. As a result, various nanoscale compounds and systems with enhanced physicochemical and functional properties in the form of simple and composite products have been developed and evaluated for this purpose in many previous studies. Among numerous nanomaterials, nanoscale metallic and non-metallic antimicrobial agents have found a wide range of applications in the medical and pharmaceutical fields by formulation or incorporation into the various products or scaffolds^13,14^. For example, various forms of Silver nanoparticles (SNPs) with different shapes, sizes, and surface functionality have been developed and studied as antimicrobial agents for application in various fields such as packaging, water disinfection, medical, and pharmaceutical fields, etc.^15,16^. The addition of SNPs in the antiseptic and disinfectant formulations, as well as their coating and deposition on the various substrates such as textiles, polymers, and metals for a wide variety of applications like deodorizing of socks, wound dressings, and biomedical devices are a few examples among others^17–20^. The antimicrobial activity of the SNPs is mainly related to the continually releasing silver ions, which disrupt the integrity of microbial cell walls, cytoplasmic membranes, and the mitochondrial respiratory chain of bacteria^21,22^.

Despite the various and unprecedented advantages of nanomaterials, the effective formulation, loading, and immobilization of these compounds into different substrates has created a new challenge in this area and restricted the application of these compounds in various fields of science and technology. Until today, different approaches have been applied to solve these problems. For example, in the study conducted by Afzal and coworkers, NPs were modified with silane-containing compounds under acidic conditions and attached to the cotton surfaces via epoxy groups under alkaline conditions^23^. However, this method involves multiple chemical reactions in different media, which impose safety concerns and higher costs to the final products. Other researchers also applied similar chemical modification procedures to develop durable nanoparticulate decorated fabrics for different applications^23–29^. Other methods in this field such as simple immersion, thin-film coating, electrospinning, and other methods based on the chemical and physical deposition of metallic vapors (e.g., CVD and PVD techniques) suffer from many drawbacks such as low efficiency, asymmetric coating, inadequate stability, coating of a limited number of metals, and uneconomic issues, etc.^30,31^. On the other hand, in biomedical applications, especially in the case of biologically harmful materials, it is highly preferred that the encapsulated or deposited compounds release continuously to provide an effective dose (ED) or effective concentration (EC) of active compounds with desired long-term activity. Controlling the release profile of these materials is essential to their effectiveness and toxicity. In addition to the positive aspects of the nano-engineered substances, their side and adverse effects have not yet been fully documented^32,33^. As a result, uncontrolled use of these substances may have detrimental and irreversible effects on human life and the environment. Therefore, it is highly crucial to develop a suitable and straightforward method for coating and incorporating these materials on different substrates with a desired amount and stability and evaluating their toxicity and side effects.

Until now, the coating of SNPs on different substrates such as polymers, textiles, paper, glass, ceramics, and other substrates by a wide variety of physically and chemically coating methods such as sol–gel method^34^, CVD^35^, electro-deposition^36^, laser-assisted immobilization^37^, sputtering^38^, etc. have been reported in previous studies. It has been shown that ultrasonic irradiation or sonochemically coating is an effective method for the in situ deposition of metallic materials on nanoscale on a wide variety of substrates, including polymers, glass, paper, and ceramics^39–42^. In this method, the in situ generations of the metal oxide (MO) nanoparticles (NPs) occur by bubble cavitation mechanism and subsequent deposition onto fabric surface by high-velocity propulsion leading to impregnation of these scaffolds. It has been demonstrated that during the cavitation process, the resulting microjets and shock waves drive NPs at very high velocities, at some point near their fusion during the inter-particle collision. These microjets can project the resulting nanoparticle toward a surface at a significant speed to cause them to adhere effectively and firmly. The sonochemical method demonstrated the homogeneous and stable coating of the SNPs with sizes ranging from 5 to 200 nm on the surface of the substrates. Depending on the substrate’s nature and other materials present in the reaction medium and ultrasonic irradiation parameters, the bond involved in the adhering of the nanoparticle to the surface could be chemical or physical. This technique results in a highly homogeneous and stable surface coating by considering and adjusting the effective parameters involved in this process^43^.

In the present study, we used the sonochemical method to fabricate antimicrobial and antiviral fabrics by depositing SNPs on non-woven Spunbond substrates at different sonication times and concentrations. The resultant fabrics have been fully characterized using Field Emission Scanning Electron Microscope (FESEM), Energy Dispersive X-Ray (EDX), X-ray diffraction (XRD), and Thermogravimetry (TGA) analysis. The mechanical properties and release profiles of coatings were evaluated by tensile and Atomic Absorption Spectroscopy (AAS) measurements, respectively. By knowing the effective antibacterial and antiviral properties of SNPs, we investigated the filtration efficiency (FE), pressure drop, and electrical conductivity of the resultant fabrics for developing facemasks suitable for different medical and general applications. The antibacterial activity of samples was also investigated by *Escherichia coli* (*E. coli*) as Gram-negative and *Staphylococcus aureus* (*S. aureus*) as Gram-positive bacteria, and cell viability was assessed using MTT and brine shrimp lethality tests using Artemia nauplii (A. salina).

## Experimental details

### Materials

Silver nitrate (AgNO_3_), Ethylene glycol, Ammonia solution (28–30%), and Ethanol were supplied from Sigma Aldrich (Germany). The Spunbond non-woven fabrics were purchased from Zanjan local market (Iran).

### Methods

#### Preparation of coated fabrics

SNPs-coated Spunbond fabrics were coated as described in the literature with some modifications^42^. Briefly, a 500 ml of water/ethanol/ethylene glycol solution (10:7:3) with different concentrations of AgNO_3_ was purged under Argon (80 mL/min) for one hour to remove the trace amount of O_2_/Air from the reaction vessel in the presence of 6 plies of fabrics. Then, the solution was irradiated under the flow of Argon at different times with a high-intensity probe sonicator (Titanium horn, 20 kHz, 400 W at 60% efficiency). After 2 min, a 28–30% Ammonia solution was introduced to the reaction medium. The temperature of the reaction was adjusted around 25–30 °C by a cooling bath. At the end of the reaction, the products were isolated and washed three times with distilled water and ethanol to remove the remaining ammonia and finally oven-dried in a vacuum oven. Different reaction conditions in terms of precursor concentrations and reaction times are used for coating the fabrics, as listed in Table 1.

**Table 1:** Different precursor concentrations and reaction times used for coating fabrics.

Reaction time (min)	Sample Code	Precursor concentration (mM)
25	1	2
	2	10
	3	20
	4	50
50	5	2
	6	10
	7	20
	8	50
Uncoated fabric	0	–

#### Characterization of fabrics

##### SEM and EDX analysis

SEM technique was used to investigate the morphology of the deposited SNPs on the Spunbond fabrics. A small section of each fabric was stuck on glass slides, placed at the SEM holder, and sputter-coated for 90 s with Gold for better conductivity during imaging. Then observations were performed using FESEM, MIRA3 TESCAN in 20.0 kV and high-vacuum mode. Elemental analysis of the coated samples was conducted using an EDX detector (EDS, MIRA3 TESCAN) attached to the FESEM machine^44^. The particle size and particle size distribution of coated samples were measured by ImageJ 1.52v software (http://imagej.nih.gov/ij/).

##### XRD analysis

To evaluate the effect of the coating process and deposited SNPs on the crystallinity of the fabrics, as well as to confirm the presence of SNPs on the fabrics, the uncoated and coated fabrics were analyzed by a wide-angle X-ray diffractometer (EQuinox 3000, INEL, France). The spectra were recorded at a scan rate of 2.4 min^-1^ in the range of 2θ = 5–80°^45^.

##### TGA analysis

The total content of SNPs on the fabrics was measured by TGA analysis (TA universal model V1.7F) by heating them from 30 to 800 °C at a heating rate of 10 °C/min under an N_2_ atmosphere. The effect of the coating procedure and coated SNPs on the fabric’s mechanical properties have also been evaluated^46^.

##### Mechanical properties

Stress–strain curves were obtained according to ASTM D8822 using a universal tensile testing machine (Santam, Iran) at room temperature. Strips with 6 × 4 cm^2^ sizes were cut off from each sample, mounted into the grips and stretched with a 10 mm/min strain rate until breakage. Two specimens of each fabric before and after coating were tested^42^.

##### Filtration efficiency and air flowability

The FE of the coated fabrics was measured by a lab-scale filtration devise against airflow and different particle sizes ranging from 0.3 to 3.0 µm and compared with uncoated samples. Briefly, samples were cut into square sheets of 10 × 10 cm^2^ and firmly fixed between two chambers of the device. By introducing airflow containing different particle sizes in one chamber, the sample’s FE and air exchange capability were measured in another chamber by a particle mass counter device^47,48^. All measurements were carried out in triplicates, and the average values were calculated.

##### Conductivity test

The conductivity of the coated fabrics was measured using a four-probe resistance IV meter. For this purpose, the coated and uncoated fabrics were cut into rectangular specimens of 20 mm in length and 10 mm in width and soaked in deionized water. Then the excess water was removed with a filter paper to perform a conductivity test in a wet state^49^. The electrical conductivity of three individual samples from each fabric was measured, and the average values were calculated.

##### In vitro release study

The content of the silver released from coated samples was measured by AAS analysis using novAA 350 (Analytik Jena, Jena, Germany). For this purpose, 2 × 2 cm^2^ of coated fabrics were accurately weighed and then placed in 50 ml distilled water as the release medium (pH 6) with a magnet bar. Then, aliquots of 5 mL of solution media were withdrawn at intervals of 24, 48, and 72 h and replaced by the same volume of freshwater. Then, the silver ion concentration in the solution was measured by the AAS method. All measurements were performed in triplicates, and the average values were calculated^50^.

##### Bactericidal assay

The antibacterial activity of coated fabrics was performed against *S. aureus* (ATCC 25,923) and *E. coli* (ATCC 25,922), as described elsewhere^42^. Both strains were obtained from the Iranian Research Organization for Science and Technology. A typical protocol was used as follows: First, the bacteria cultures were prepared using nutrient agar modified (QUELAB QB-39-3504) overnight. Then, the cultures were transferred into a nutrient broth (NB) and incubated at 37 °C with aeration. After reaching the logarithmic phase, they were centrifuged and washed to yield a final bacterial concentration of approximately 10^8^ CFU ml^−1^. Next, 500 μL of the strain cells containing 10^8^ CFU ml^−1^ of each strain was transferred into a vial containing 4.5 ml of a saline solution and coated fabrics (1 cm × 1 cm). Besides test samples, saline without the sample and saline with an uncoated sample was included in the experiment as control groups. After incubation of bacterial suspensions at 37 °C for four hours, 100 μl of each sample at specific time intervals (t = 0, 1, and 3 h) was transferred onto nutrient agar plates after dilution with saline. The plates were incubated overnight at 37 °C, and viable bacteria were counted. The number of initial CFU (N0) and final CFU at the specific times (N) were used to determine the bacterial survival fraction (N/N_0_). The antibacterial activity of fabrics were performed in triplicates, and the average values were calculated.

##### MTT assay

The cytotoxicity effect of silver-coated fabrics was conducted by MTT assay as described by Vijayakumar^51^. Briefly, 1 × 10^5^ mL^−1^ cells (HEK 293) were seeded in a 96-well plate in their exponential growth phase and were incubated overnight at 37 °C and 5% CO_2_ in a humidified atmosphere. The solutions of released silver at 24, 48, and 72 h (in vitro release test section) were added to the plate and incubated for 24 h. Then, 10 μL of MTT reagent was transferred to each well and was further incubated at 37 °C for four hours to reduce tetrazolium to insoluble formazan. After formazan crystals were dissolved in DMSO, the plates were read immediately at 545 nm in a microplate reader (infinite M200, TECAN). Wells with complete medium and MTT reagent without cells were used as blanks. Untreated HEK 293 cells and the cell treated with each concentration of SNPs for 24, 48, and 72 h were subjected to the MTT assay for cell viability determination. The lethality was calculated using Abbott’s formula as follows: % Lethality = [(Test − Control)/Control] × 100. All measurements were conducted in quintuplicate, and the average values were calculated.

##### Toxicity testing by A. salina

The cytotoxicity evaluation of silver-coated fabrics by the A. salina method was performed as described by Rajabi et al.^52^. A. salina eggs were purchased from the Aquatic Animal Research Center, Urmia University, Urmia, Iran. Briefly, dried cysts were transferred into a bottle containing artificial seawater prepared by dissolving 35 g of NaCl in one liter of distilled. Then, the bottle containing A. salina eggs was incubated at room temperature (RT) under strong aeration and continuous illuminations^33^. After the larvae (nauplii) hatched, 200 μL of silver solutions with different concentrations were added to each well of the 96-well microtiter plates. Then, 10 nauplii per well were added to each well and incubated at RT for 24, 48, and 72 h. After 24 h, the numbers of surviving nauplii in each well were counted under a stereoscopic microscope. The experiments were conducted in quintuples for each solution. The negative control wells contained 10 nauplii and artificial seawater only. All measurements were performed in quintuplicate, and the average values were calculated.

##### Statistical analysis

The statistical analysis was assessed by Student's t-test or One-Way ANOVA. *P* < 0.05 indicated the statistical significance. The form of mean ± standard deviation (SD) was used to convey the data. The data analysis software was SPSS v26 (IBM SPSS Statistics, Chicago, IL, USA).

## Results and discussion

**Figure 1:**
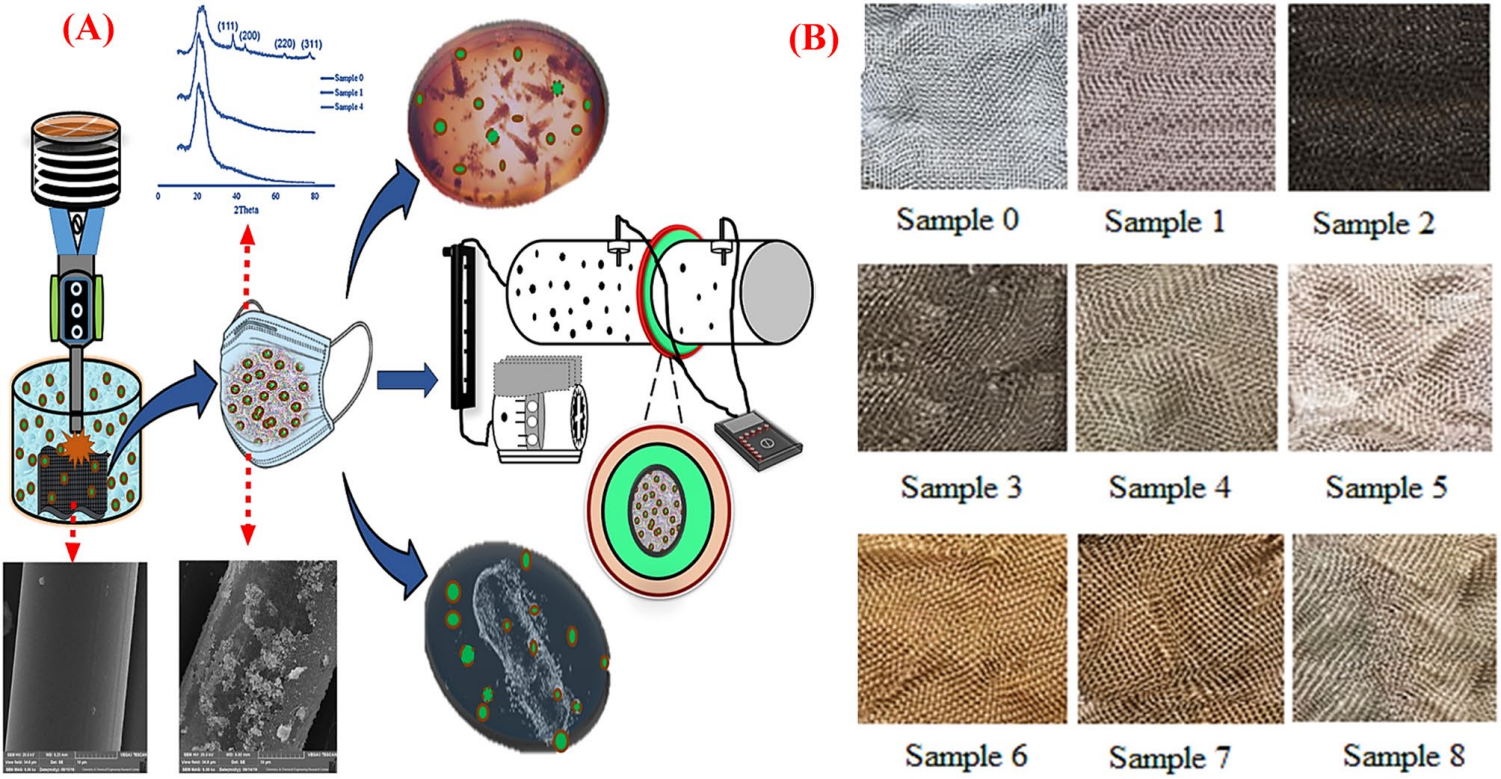
(**A**) Schematic illustration of the preparation of sonochemically-coated fabrics to develop antibacterial and antiviral facemasks. (**B**) Uncoated and SNP-coated fabrics by sonochemically coating procedure. Sample 0: Uncoated fabric, Samples 1–4 and 5–8: NS-coated fabrics at 2, 10, 20, and 50 mM silver nitrate concentrations, and 25 and 50 min sonication time, respectively.

**Figure 2:**
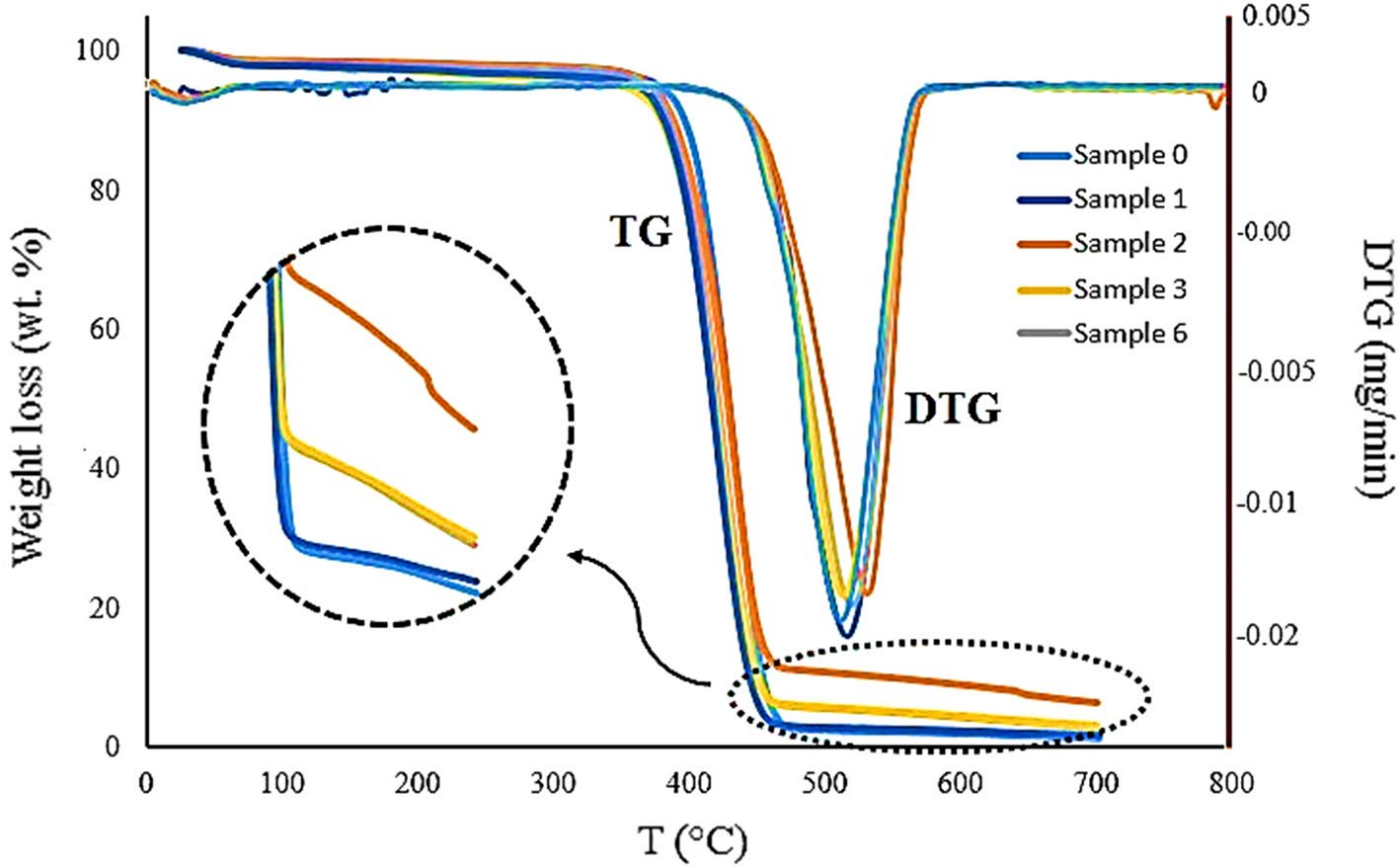
TGA curves of different coated Spunbond fabrics. Sample 0 (Uncoated fabric), Samples 1, 2, 3 (NS-coated fabrics at 2, 10, and 20 mM silver nitrate concentrations, and 25 min sonication time, respectively), Sample 6 (NS-coated fabrics at 10 mM silver nitrate concentration and 50 min sonication time).

In this study, we coated Spunbond fabrics with SNPs at different concentrations and times and evaluated their physicochemical and functional properties to fabricate antibacterial and antiviral facemasks (Fig. 1A). Ethylene glycol was used as a reducing agent for producing SNPs and subsequent deposition on the fabrics. In this process, the [Ag(NH_3_)_2_]^+^ complex formation takes place by the addition of ammonia solution to the reaction medium. Because of the large constant equilibrium of the formation of this complex, the concentration of silver ions remains in a small amount in equilibrium with the complex. Therefore, the ammonia concentration and Ag^+^/NH_3_ ratio in the reaction medium is an important factor in reducing the silver ions to the desired particle size. As a result, in this study, the ratio of Ag^+^/NH_3_ was kept constant, and only the effect of different concentrations of silver and reaction times were evaluated for obtaining the efficient coating of SNPs on the fabrics. The images of all uncoated and coated fabrics are depicted in Fig. 1B. Different concentrations of silver precursor in the reaction medium and different coating times significantly affect coating efficiency, which is obvious from the color of the coated fabrics. Obviously, by increasing the silver concentration and reaction time, the color of fabrics turned darker because of the higher loading of SNPs on the fabrics. The deposited silver content on the fabrics for different precursor concentrations (Samples 0, 1, 2, and 3) and different times (Samples 0, 2, and 6) was determined by TGA analysis and illustrated in Fig. 2. From respective TGA diagrams for each sample, it is evident that the content of deposited silver on the surface of the fabrics depends on the silver nitrate concentration and reaction time. The weight percent of deposited silvers varies from 0.1%, 3.2%, 3.4% and 8.5% for 1, 3, 6 and 2 samples, respectively.

**Figure 3:**
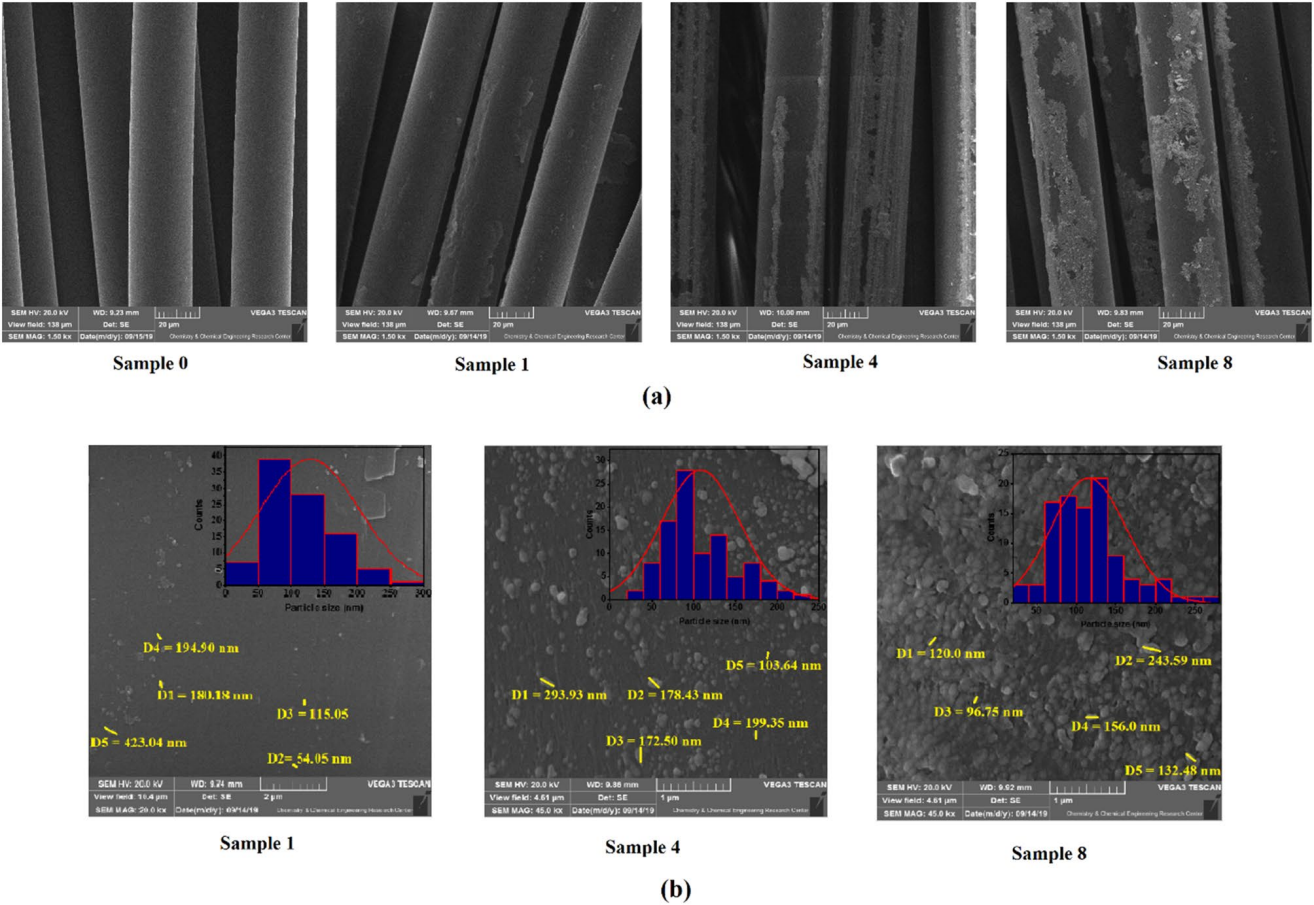
SEM images of coated and uncoated samples. (**a**) Deposition of SNPs on fabrics as a function of precursor concentration and sonication time, (**b**) Particle size and particle size distribution of deposited SNPs. Sample 0 (Uncoated fabric), Samples 1 and 4 (NS-coated fabrics at 2 and 50 mM silver nitrate concentrations, and 25 min sonication time, respectively), Sample 8 (NS-coated fabrics at 50 mM silver nitrate concentration and 50 min sonication time).

**Figure 4:**
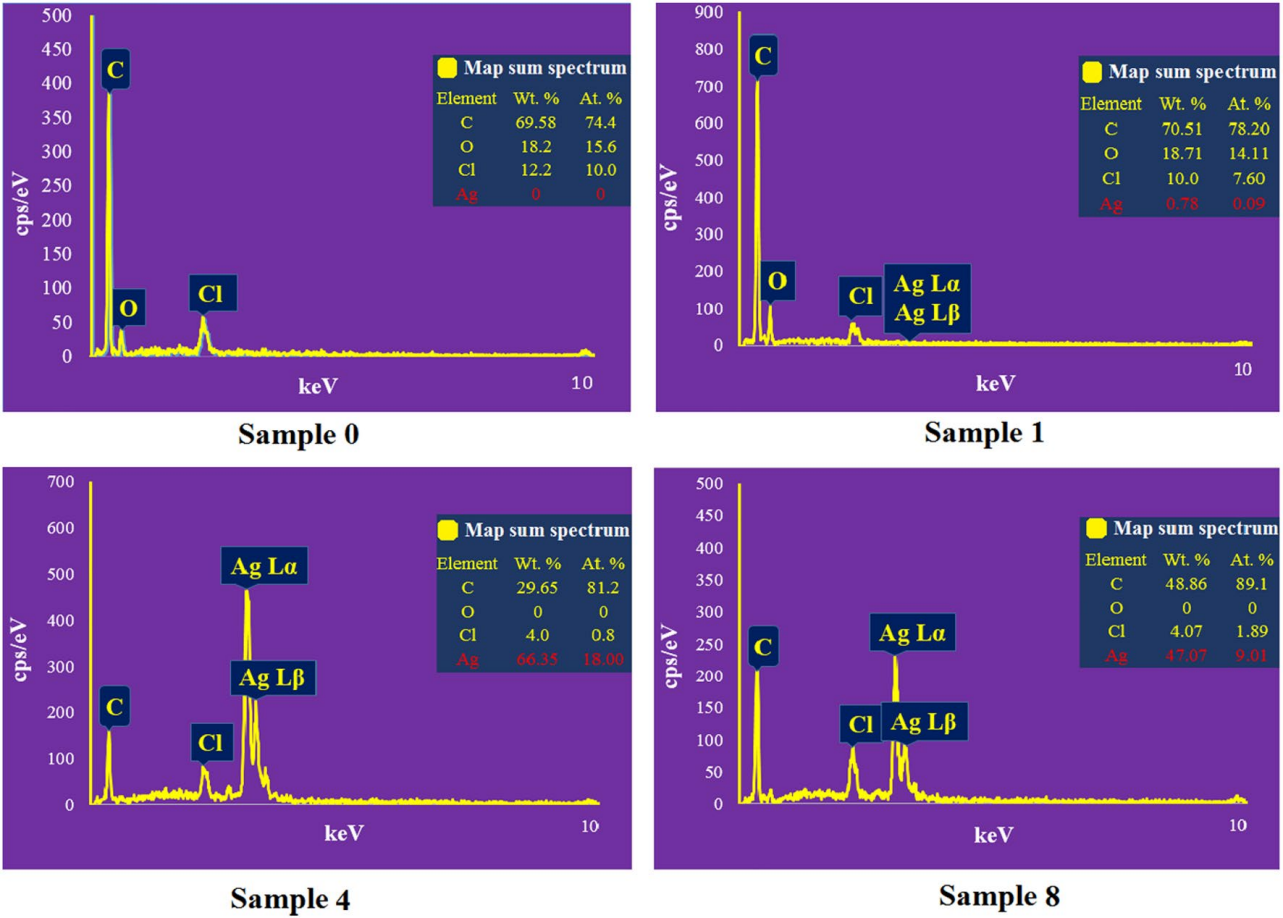
EDX spectra of the uncoated and coated samples indicating the presence of SNPs on the coated fabrics. Sample 0 (Uncoated fabric), Samples 1 and 4 (NS-coated fabrics at 2 and 50 mM silver nitrate concentrations, and 25 min sonication time, respectively), Sample 8 (NS-coated fabrics at 50 mM silver nitrate concentration and 50 min sonication time).

The morphological characteristics of the samples were investigated by the SEM technique (Fig. 3). The SEM images in Fig. 3a support the results obtained from the TGA analysis. It was shown that the content of deposited silver on the fabrics is in direct correlation with the precursor concentration and reaction time. With increasing the precursor concentration from 2 mM (sample 1) to 50 mM (sample 4) and reaction time from 25 min (sample 4) to 50 min (sample 8), the content of deposited silver on the fabrics was increased. However, coated sample 2 showed unusual results with the highest SNPs coating over all samples. This figure also highlights the differences between coated (Samples 1, 4, and 8) and uncoated fabrics (Sample 0). Figure 3b shows the particle size and particle size distribution of coated samples measured by ImageJ software. The particle size of the deposited silver on the fabrics is on the nanoscale. It is evident that increasing the precursor concentration and sonication time results in the formation of larger particles and higher deposition of NSs (100.14 ± 8, 105.97 ± 14, and 125.12 ± 21 nm for samples 1, 4, and 8, respectively). On the other hand, the particle size distribution was increased with increasing sonication time. These results are in good agreement with previous studies where other precursors and substrates were coated with the ultrasonication method^42,53^. EDX spectra recorded from the uncoated and coated samples are shown in Fig. 4. It is clear that the coated fabrics have the weight percentage of silver as 0.78%, 66.35%, and 47.07% for 1, 4, and 8 samples, respectively. These results again indicate the increases in the silver deposition on the fabrics by increasing the precursor concentration and reaction time.

**Figure 5:**
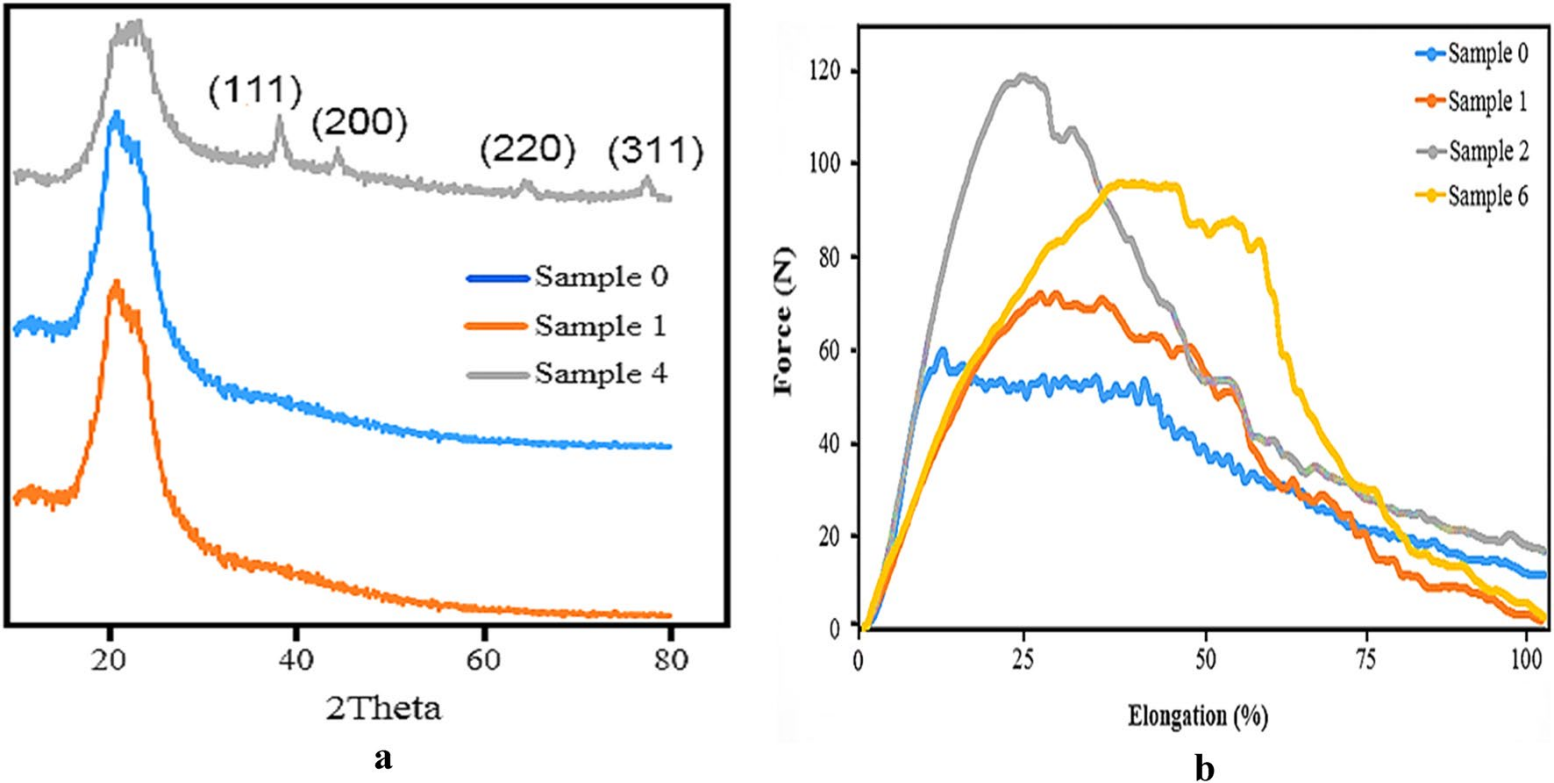
XRD patterns (**a**) and mechanical properties (**b**) of the uncoated and coated fabrics. Sample 0 (Uncoated fabric), Samples 1, 2, 4 (NS-coated fabrics at 2, 10, and 50 mM silver nitrate concentrations, and 25 min sonication time, respectively), Sample 6 (NS-coated fabrics at 10 mM silver nitrate concentration and 50 min sonication time).

XRD measurements of coated and uncoated fabrics have been shown in Fig. 5a. Uncoated sample 0 and coated sample 1 did not show any characteristic diffraction peaks of silver at all 2-theta scales. Coated sample 1 showed a very low silver content (0.1% from TGA analysis), so the characteristic diffraction peaks of silver are absent in the XRD pattern of this sample. On the other hand, the coated sample 4 showed the characteristic diffraction peaks of silver at 2θ values of 38.1°, 44.45°, 64.55°, and 77.40° corresponding to the (111), (200), (220), and (311) planes of metallic silver, respectively^54,55^. The mechanical properties of coated and uncoated samples were also evaluated to determine the effect of the coating procedure on the mechanical behavior of samples (Fig. 5b). As shown in the figure, silver-coated fabrics (Samples 1, 2, and 6) show a somewhat more brittle behavior than the uncoated fabric (Sample 0). This behavior was higher for coated fabrics with higher concentrations of precursors at the same reaction time (Sample 2 and 6 vs. sample 1) and longer reaction times at the same precursor concentration (Sample 2 vs. Sample 6). On the other hand, the tensile force for the coated samples 1, 2, and 6 was surprisingly about 27%, 55%, and 46% higher than the uncoated sample, respectively. These results are in direct correlation with precursor concentration and reaction time. However, previous studies have shown both consistent and inconsistent results with the results obtained in this study. In some of these studies, the coated samples showed less tensile force than uncoated samples^42^. However, the other studies corroborated the result of the present study as the coating process has led to an increase in the tensile force. The latter is attributed to the enlargement of the fiber diameter due to the ductile nature of silver metal^56^.

**Figure 6:**
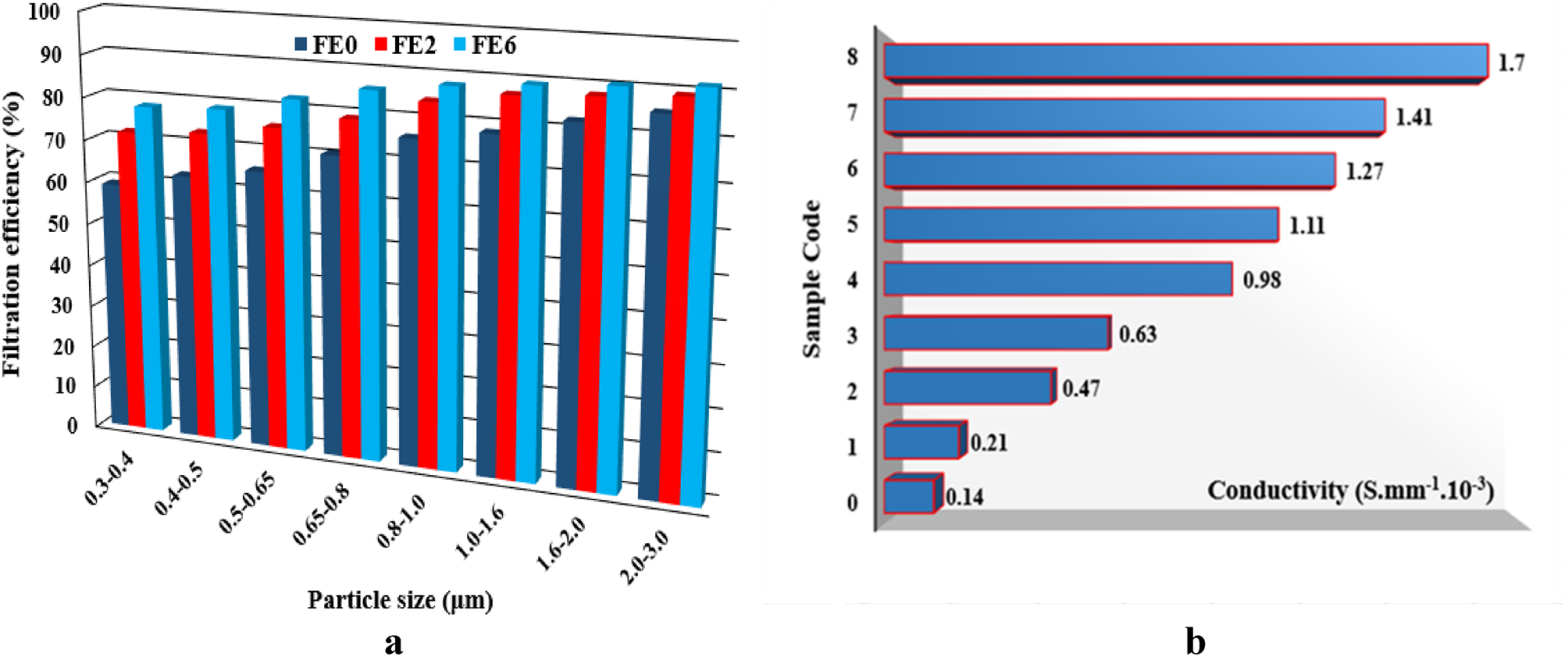
The FE of the coated samples against particles of different sizes (**a**) and the electrical conductivity of the fabrics (**b**). Sample 0 (Uncoated fabric), Samples 1–4 and 5–8 (NS-coated fabrics at 2, 10, 20, and 50 mM silver nitrate concentrations, and 25 and 50 min sonication time, respectively).

Evaluation of the drop pressure of the fabrics resulted in the air flowability of 30 LPM for both coated and uncoated samples, which support normal and easy breath^57–59^. On the other hand, the FE of the coated samples confirmed the adequate performance of the resultant fabrics in the filtration of particles of different sizes (Fig. 6a). The uncoated sample showed a 52.75–76.88% FE for particle sizes in the range of 0.3 to 3.0 µm, while samples 2 and 6 resulted in 78.94–89.32% and 81.24–91.60% FEs for particles in this size range, respectively. These results are significantly higher than the values reported for many commercial and handmade facemasks and comparable to N95 masks that are currently used by the public^60–64^. On the other hand, the fabricated facemasks in the present study possess additional advantages by providing antibacterial activity and improved drop pressure compared with N95 masks. Increasing the FE by deposition of SNPs attributed to the increase in the tortuosity of the fabrics by filling the open and connected voids. PP meltblown fabrics were commonly used to fabricate traditional and medical facemasks and gowns. The FE of these products relies on their static electricity originating from the meltdown process. However, the efficacy of electrostatic filtration drops drastically in the moist environment and by prolonged use or re-use. Moreover, smaller particles such as bacteria and virions can easily pass through these fabrics due to their larger pore diameter. In this regard, the study conducted by Dnyanmote et al. indicated that the diameter of the aerosol-carrying virus is bigger than 2.5 µm, and a membrane with a pore diameter of 0.18–0.5 µm could provide a highly effective filtration. Finally, they concluded that the doping or impregnating these scaffolds with metallic NPs like SNPs could enhance the FE of the resultant fabrics against COVID 19^65^. Besides the positive effect on the FE of the fabrics, other studies assessed the antiviral activity of SNPs against coronavirus. For example, Magomedow et al. examined the possible mechanisms by which SNPs could influence coronavirus using computer quantum-chemical modeling. The results revealed that the formation of the "tryptophan-SNPs" complex (E = -5856.83 kcal/mol) is the most energy-efficient interaction among other possible interactions. Moreover, the most stable complex was the "cysteine-SNPs" complex (Delta E = 0.16 a.u.). Therefore, they concluded that SNPs could exert antiviral activity through the interaction with tryptophan and cysteine amino acids of the coronavirus spike proteins^66^. Other studies also reported similar results about SNP’s antiviral activity^67–69^. These findings clearly indicate the potential application of the coated samples in developing efficient facemasks or gowns for different situations like the COVID-19 pandemic and other medical and industrial applications.

The electrical conductivity of the fabrics was examined using a four-probe resistance meter in the wet state, and the results are depicted in Fig. 6b. As we can see, the conductivity of the fabrics increases by increasing the content of SNPs deposited on fabrics. In this respect, samples 4 and 8 showed conductivity of 1.7 × 10^–4^ S∙mm^-1^ and 0.98 × 10^–4^ S∙mm^-1^, as a result of higher precursor concentration and sonication time, respectively. It is worth mentioning that the electrical conductivity of sample 8 is nearly 12-times compared to uncoated fabrics. On the other hand, the results clearly indicate that the fabrics possess high conductivity in the wet state, which is highly beneficial for different applications. As noted above, the electrostatic properties of the PP meltblown fabrics play an important role in providing effective FE against environmental and biological pollutants. However, these fabrics significantly lose their effectiveness upon contact with water or a moist environment. In this regard, major attempts have been made to fabricate electroceutical fabrics that maintain an electric field in a moist environment or wirelessly generate a low level of electricity in the presence of moisture. For example, Ghatak et al. fabricated an electroceutical polyester fabric printed with alternating circular regions of Ag and Zn dots and evaluated its performance in deactivating porcine respiratory coronavirus AR310 particles. The results showed that the resultant fabrics significantly lower the zeta potential of the virion in one minute of contact and lead to the eradication of its infectivity through destabilization of its electrokinetic properties and subsequent aggregation^70^. The electroceutical fabrics are also expected to find potential applications in other fields, such as wound healing^71–73^.

**Figure 7:**
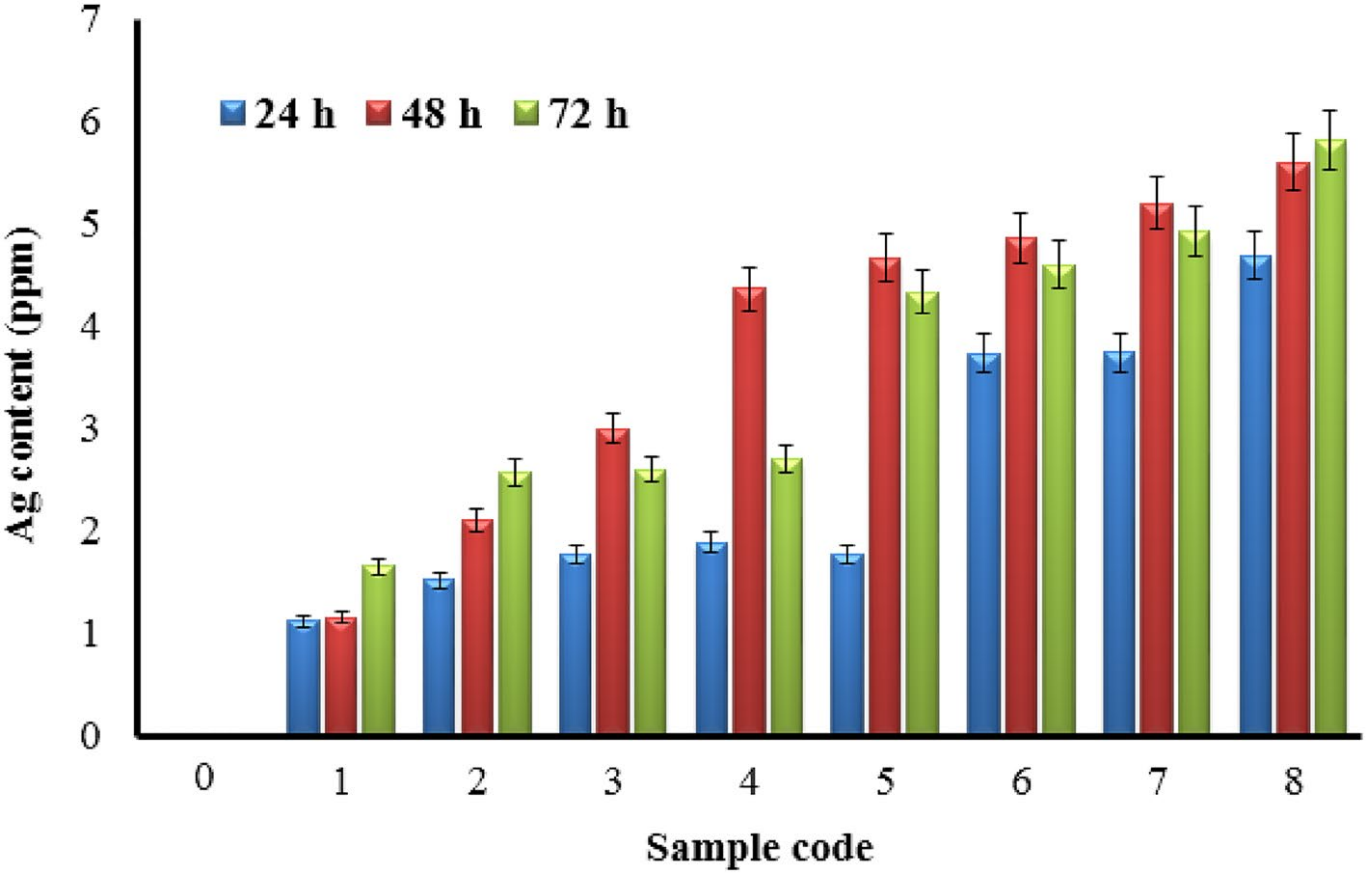
In vitro release of deposited SNPs from coated (Samples 1–8) fabrics. Uncoated fabric (Sample 0) included as blank in the experiment. Sample 0 (Uncoated fabric), Samples 1–4 and 5–8 (NS-coated fabrics at 2, 10, 20, and 50 mM silver nitrate concentrations, and 25 and 50 min sonication time, respectively).

**Figure 8:**
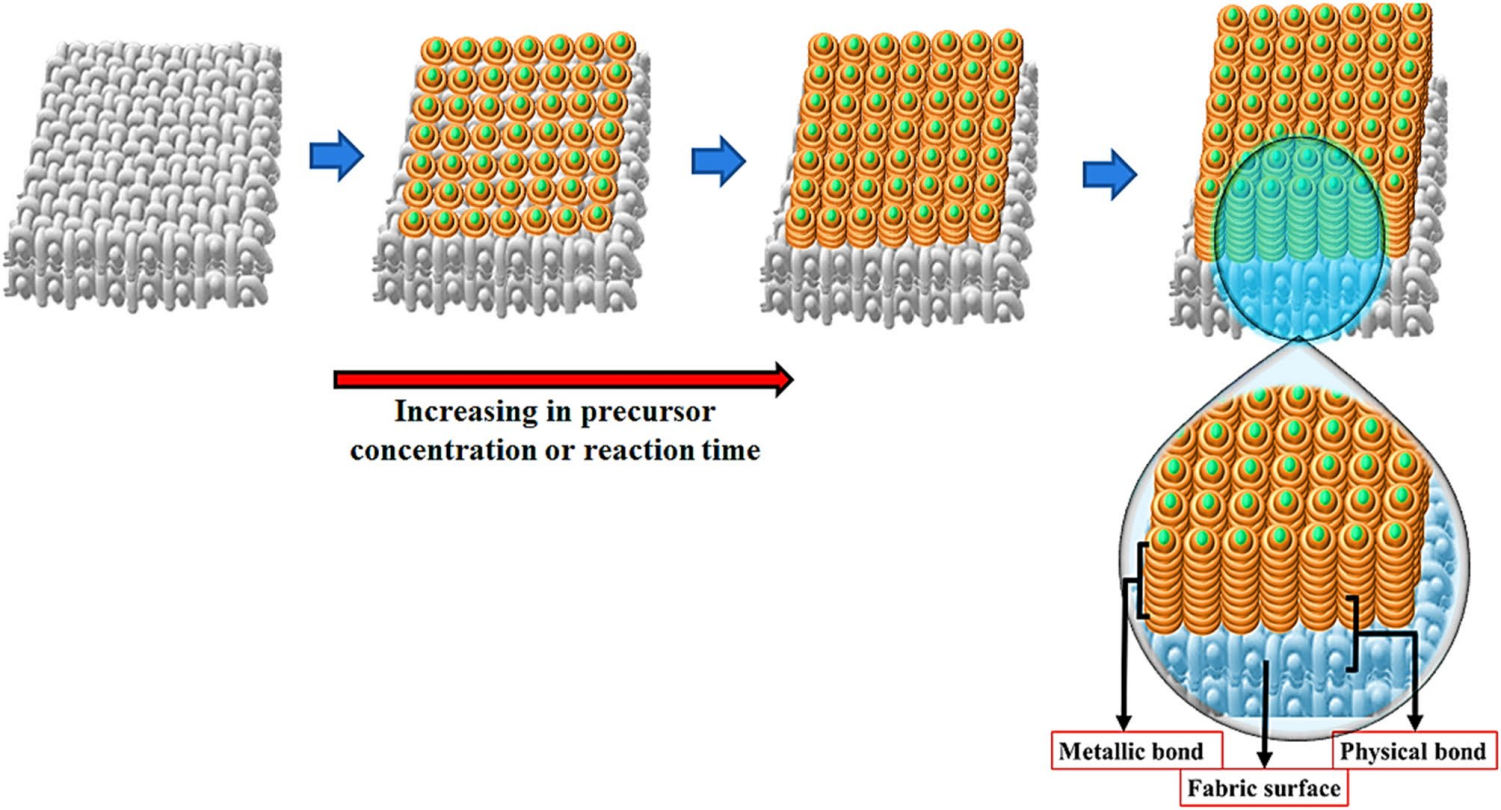
Schematic representation of SNPs bonds on fabrics as a function of precursor concentration and reaction time.

In the next step, the coated and uncoated fabrics were subjected to in vitro release study for evaluation of the coating’s stability. The 2 × 2 cm^2^ specimens were immersed in 50 ml distilled water with a magnet bar, and 5 ml of the resultant silver-containing solutions were subjected to AAS analysis after 24, 48, and 72 h intervals to determine the released content of silver. Referring to Fig. 7, the in vitro accumulative release of SNPs shows a relatively time-dependent behavior over 72 h. However, regarding the cumulative nature of the study and relatively constant increase in silver content at specific time intervals, it can be concluded that the release profile of silver has been constant over time. It is evident that the accumulative release content decreases with increases in the reaction time (Samples 1–4 vs. Samples 5–8) and increases with increasing the precursor concentration (Samples 1 to 4 and Samples 5 to 8). In other words, higher precursor concentrations and reaction times lead to strong adhesion of the NPs to the fabric surface, suggesting a more stable coating. However, more SNPs were deposited on the fabrics in higher precursor concentrations and reaction times, resulting in a higher amount of silver released into the medium. In a typical sonochemical process, in situ generations of NPs occurs in the reaction medium and follows by simultaneous adhering to fabrics by ultrasonically generated fluidic microjets. Anchoring of metal NPs to the fabric surface by physical, chemical, or other interactions in the sonochemical process has been reported in previous studies^74^. However, according to the present study results, it seems that the nature of metallic NP bindings to the surface of the fabrics is of a physical type (Fig. 8). First, a metallic monolayer covers the surface by physically anchoring the SNPs to the fabric surface. Next, metallic NPs deposit layer-by-layer (hypothetical) on the fabric surface by strong metal bonds. Because higher sonication times lead to more silver deposition, stronger binding of NPs could be achieved for these samples, resulting in a lower amount of silver released from fabrics. On the other hand, the strong deposition of SNPs in longer reaction times somehow enhances the interaction of the first layer with the fabric surface. Physical bonding between the fabric surface and the metal or metal oxide NPs rather than chemical bond formation was also reported by Perelshtein et al.^75^.

**Figure 9:**
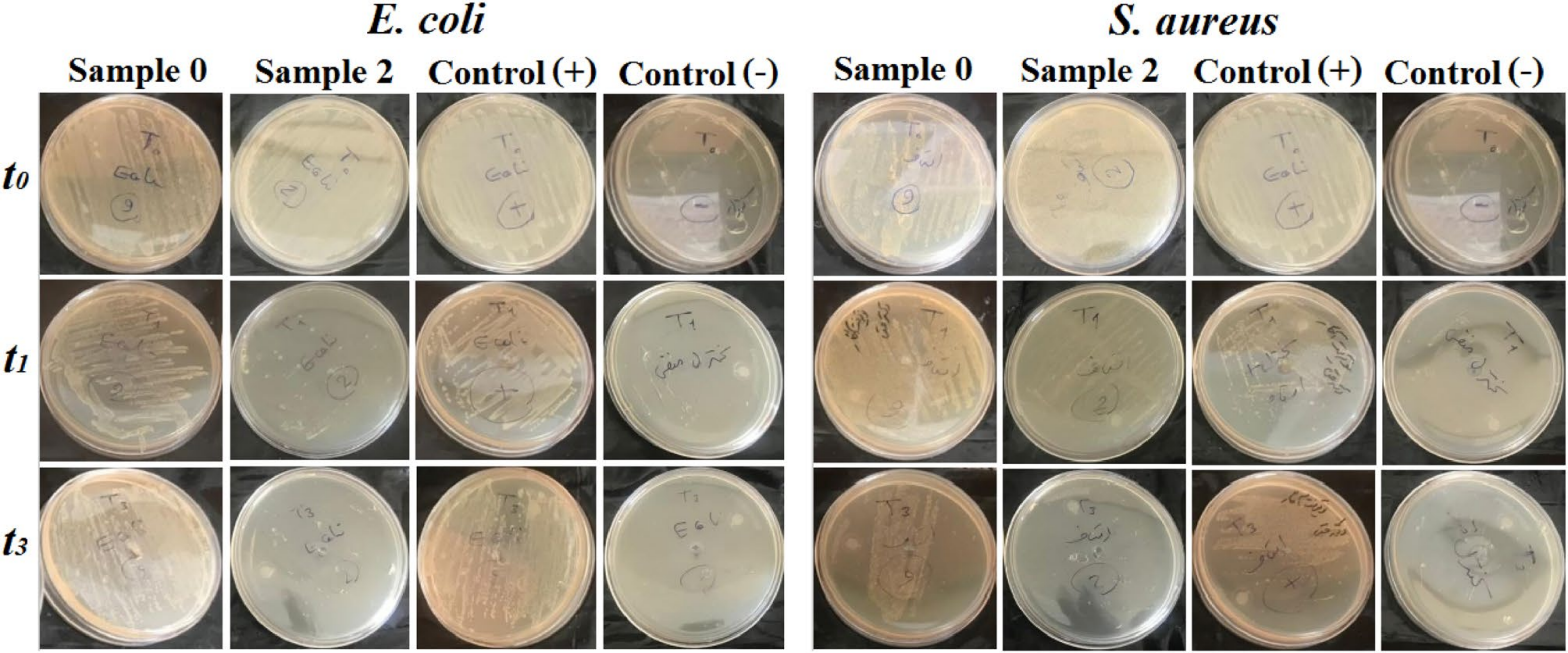
Antibacterial activity of coated fabric (Sample 2, NS-coated fabrics at 2, 10, 20, and 50 mM silver nitrate concentrations, and 25 min sonication time) in comparison with uncoated fabric (Sample 0, uncoated fabric) at different experiment time. Control ( +): saline without sample, Control (−): saline with uncoated sample and without bacteria.

The minimum inhibitory concentration (MIC) and minimum bactericidal concentration (MBC) of SNPs were reported as 3.12 ppm and 6.25 ppm for *E. coli*, and 0.625 ppm (both MIC and MBC) for *S. aureus*, respectively, which are in the range of silver amounts released form coated fabrics (1–6 ppm)^76,77^. Therefore, in the next step the antibacterial activity of SNP-coated fabrics was carried out against *S. aureus* and* E. coli as a* Gram-positive and Gram-negative bacteria. The results have been summarized in Table 2 and depicted in Fig. 9. Efficient antibacterial activity against both bacterial strains was observed for all coated fabrics. As shown in Table 2, three hours of incubation led to total inhibition of both bacteria, while only in the case of *E. coli*, 100% bactericidal activity was observed within the first hour. However, a 100% reduction for *S. aureus* was achieved only after 3 h of incubation. This difference in the results between Gram-negative and Gram-positive bacteria is attributed to the SNPs bactericidal mechanism and differences in the cell membrane structure of these two strains of bacteria. According to the generally accepted mechanism, SNPs continuously release silver ions that attach to bacteria’s cell wall and cytoplasmic membrane through electrostatic interactions and high affinity to sulfur proteins. Thereby, increasing the membrane permeability and changing the cell functionality eventually leads to cell death^78–80^. On the other hand, Gram-positive and Gram-negative bacteria are very different in structure, morphology, and cell wall components. For example, due to the high thickness of the cell wall and dense peptidoglycan layer, the Gram-positive bacterias show more protection against antibacterial agents than Gram-negative bacteria, which are mostly made of tightly packed lipopolysaccharides (LPS). These differences can lead to differences observed in antibacterial testing for these two types of bacteria. However, as shown here, fabrics coated with SNPs are effective for both groups of these bacteria.

**Table 2:** Antibacterial activity test using *E.coli* and *S. aureus*. The viable bacteria were monitored by counting the number of colony-forming units (CFU).

Sample code	Duration of treatment (h)								
	*t*_*0*_			*t*_*1*_			*t*_*3*_		
	CFU ml^-1^	N/N_0_	Reduction in viability (%)	CFU ml^-1^	N/N_0_	Reduction in viability (%)	CFU ml^-1^	N/N_0_	Reduction in viability (%)
*E. coli*									
0	1.5 × 10^8^	1	0	1.5 × 10^8^	0	100	1.5 × 10^8^	0	0
1	1.5 × 10^8^	1	0	0	–	100	0	–	100
2	1.5 × 10^8^	1	0	0	–	100	0	–	100
3	1.5 × 10^8^	1	0	0	–	100	0	–	100
4	1.5 × 10^8^	1	0	0	–	100	0	–	100
5	1.5 × 10^8^	1	0	0	–	100	0	–	100
6	1.5 × 10^8^	1	0	0	–	100	0	–	100
7	1.5 × 10^8^	1	0	0	–	100	0	–	100
8	1.5 × 10^8^	1	0	0	–	100	0	–	100
*S. aureus*									
0	2 × 10^8^	1	0	2 × 10^8^	0	0	2 × 10^8^	0	0
1	2 × 10^8^	1	0	1.5 × 10^7^	7.5 × 10^–2^	92.50	0	–	100
2	2 × 10^8^	1	0	1 × 10^5^	5 × 10^–4^	99.95	0	–	100
3	2 × 10^8^	1	0	2 × 10^5^	1 × 10^–3^	99.90	0	–	100
4	2 × 10^8^	1	0	1 × 10^6^	5 × 10^–3^	99.50	0	–	100
5	2 × 10^8^	1	0	2 × 10^6^	1 × 10^–2^	99.00	0	–	100
6	2 × 10^8^	1	0	1.5 × 10^6^	7.5 × 10^–3^	99.25	0	–	100
7	2 × 10^8^	1	0	1.5 × 10^6^	7.5 × 10^–3^	99.25	0	–	100
8	2 × 10^8^	1	0	2 × 10^6^	1 × 10^–2^	99.00	0	–	100

N/No: survival fraction, Sample 0 (Uncoated fabric), Samples 1–4 and 5–8 (NS-coated fabrics at 2, 10, 20, and 50 mM silver nitrate concentrations, and 25 and 50 min sonication time, respectively).

**Figure 10:**
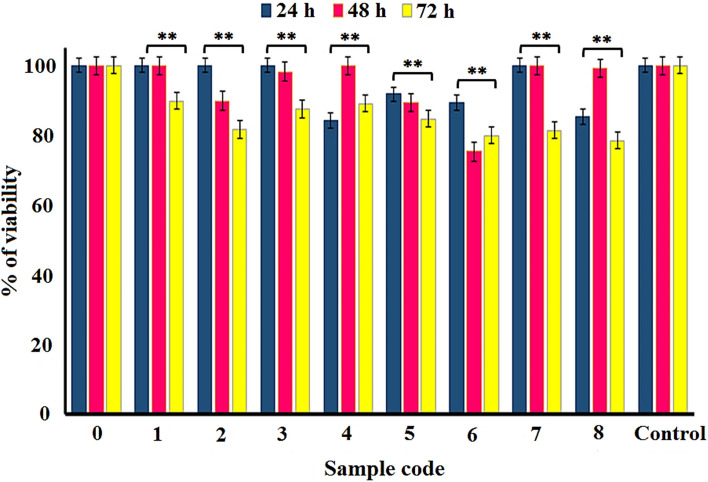
Comparison of cytotoxicity of uncoated and coated fabrics towards HEK 293 cells after 24, 48, and 72 h of exposure, **P < 0.05. Sample 0 (Uncoated fabric), Samples 1–4 and 5–8 (NS-coated fabrics at 2, 10, 20, and 50 mM silver nitrate concentrations, and 25 and 50 min sonication time, respectively).

The cytotoxic effects of the coated fabrics were evaluated by MTT assay by exposing HEK 293 cell lines to different concentrations of the SNPs solutions obtained from in vitro release study at different time intervals (Fig. 10). As shown in Fig. 10, all coated samples showed high safety over test times. For all samples, the viability percentage of HEK 293 cells decreased in a time-dependent manner at 24 h, 48 h, and 72 h, indicating that with increasing the release time, a higher amount of SNPs are released from fabrics. On the other hand, the cell viability in samples 1–4 was higher than in samples 5–8, which is reasonably attributed to a lower amount of silver deposited on the fabrics and subsequently released into the medium. From this point of view, the viability percent of HEK 293 cells also exhibits a concentration-dependent manner. In other words, cell viability has decreased by increasing the SNPs liberation, which, in turn, depends on the initial silver precursor concentration and sonication time. Therefore, we can conclude that higher sonication time results in sturdy and stable nanomaterial coatings.

**Figure 11:**
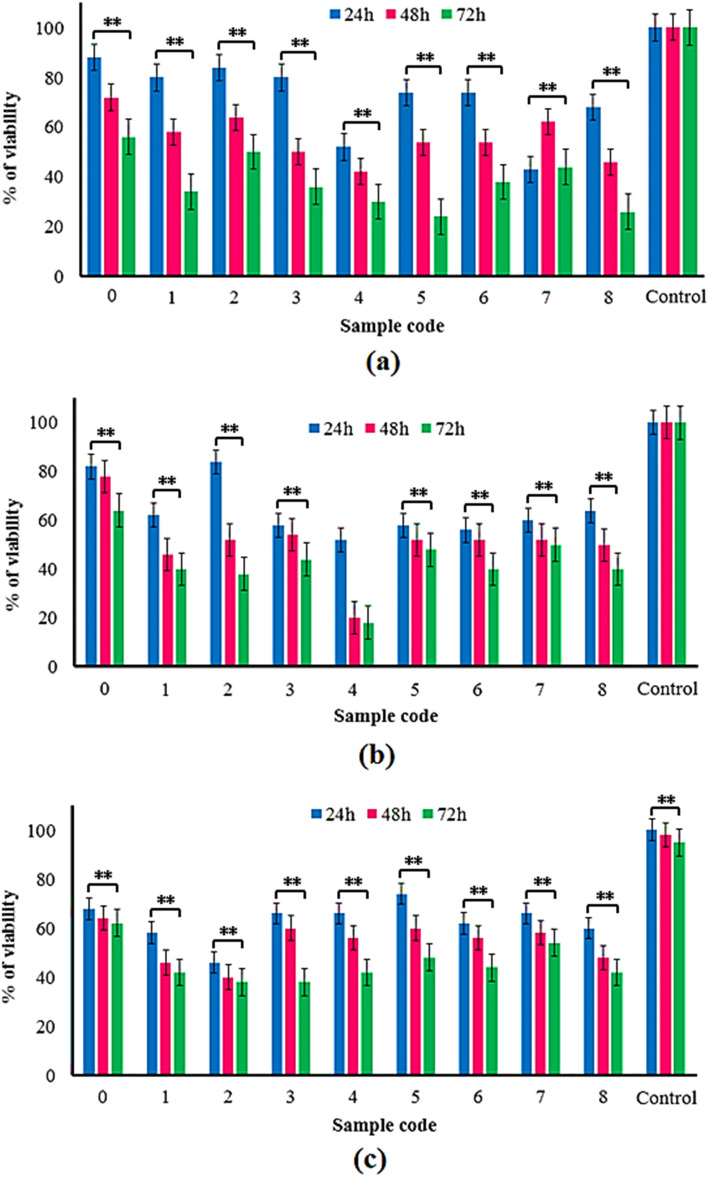
Bar diagram shows results for mortality rate (a = 24, b = 48, and c = 72 h) of brine shrimp A. Salina treated with solutions obtained from released SNPs from coated fabrics at 24, 48, and 72 h in comparison with uncoated fabric as blank, **P < 0.05. Sample 0 (Uncoated fabric), Samples 1–4 and 5–8 (NS-coated fabrics at 2, 10, 20, and 50 mM silver nitrate concentrations, and 25 and 50 min sonication time, respectively).

The brine shrimp lethality assay was also conducted to evaluate the cytotoxicity of released SNPs from coated fabrics. Figure 11 shows similar results obtained from the MTT cytotoxicity test. Therefore, the viability percentage of A. Salina decreases in a concentration-dependent and time-dependent manner. In other words, with increasing the test time, the survival rate of A. Salina has declined. In addition, with increasing the concentration of released SNPs during 24–72 h, the survival rate of A. Salina has been decreased. Again, we can see that the viability percentage of the A. Salina population in samples 1–4 is higher than in samples 5–8. As mentioned above, stable SNPs coatings occur in the samples prepared in higher precursor concentrations and sonication time. However, the amount of deposited SNPs is also higher in these samples, which results in a higher amount of silver release and reduced viability of A. Salina. Therefore, the lower amount of deposited SNPs in these samples (1–4) resulted in an unexpected more A. Salina surviving rate.

## Conclusion

The present study demonstrates the sonochemically coating of SNPs on the surface of Spunbond fabrics in different precursor concentrations and reaction times for the development of antibacterial facemask fabrics. The results revealed that this one-step and straightforward method results in remarkably stable coatings by varying the effective parameters that control the extent and quality of coatings on fabrics. The results also showed that the content of deposited SNPs on the fabrics increases with increasing the precursor concentration and reaction time. On the other hand, increasing the sonication time and precursor concentration has increased the particle size and particle size distribution of deposited silver. Further investigation of the coated fabrics in the in vitro release study revealed that higher reaction time results in stable and sturdy coatings and lower content of released silver. Physical bonding between the SNPs and fabrics and metallic bonding between SNPs in a higher amount of deposited silver are speculated for these findings. The particulate-induced FE and enhanced electrical conductivity of the coated textiles provided suitable clearance of particles of different sizes and offer desired air flowability characteristics. The coated fabrics also demonstrated highly efficient antibacterial activity against Gram-positive and Gram-negative bacteria. Investigation of cytotoxicity effects of coated fabrics by MTT assay and brine shrimp lethality test showed high safety for HEK 293 cells and Artemia nauplii. However, the higher concentration of precursor resulted in higher cytotoxicity effect in Artemia nauplii test via high rate of drug release, which should be considered and resolved in future studies. Consequently, it is revealed that a higher concentration of silver precursor and sonication times leads to a strong and uniform coating of SNPs, lower liberation of silver from coated fabrics, and higher antibacterial activity and safety for these fabrics. However, additional studies are required to assess the in vitro and in vivo antiviral activity of the coated samples. In the end, we think that the sonochemically coating technique could be applied for preparing products with enhanced properties and applications, including air filtration, antibacterial applications, food packaging, textile industries, etc.

## Data Availability

All data generated or analysed during this study are included in this published article.
